# C-peptide Level in Patients With Uncontrolled Type 2 Diabetes Mellitus on Oral Anti-diabetic Drugs

**DOI:** 10.7759/cureus.56810

**Published:** 2024-03-24

**Authors:** Purnendu Arya, Noor Husain, Chakrapani Kumar, Ravi Shekhar, Ved Prakash, Saajid Hameed, Lalit Mohan, Harihar Dikshit

**Affiliations:** 1 Department of Pharmacology, Indira Gandhi Institute of Medical Sciences, Patna, IND; 2 Department of Biochemistry, Indira Gandhi Institute of Medical Sciences, Patna, IND; 3 Department of Endocrinology, Indira Gandhi Institute of Medical Sciences, Patna, IND

**Keywords:** hypoglycemic agents, insulin, glycemic control, c-peptide, diabetes mellitus type 2

## Abstract

Background: In the development and progression of type 2 diabetes mellitus, β-cell dysfunction occurs after insulin resistance. Despite poor glycaemic control, there is a practice of increasing the dose of oral anti-diabetics or adding more drugs to the regimen due to the common perception that low endogenous insulin secretion is related to type 1 diabetes mellitus only and patient's poor compliance to injectables. Keeping this perspective in mind, this study was conducted to assess the prevalence of beta cell dysfunction by low serum C-peptide levels and its correlation with poor glycaemic control.

Materials and methods: A total of 134 patients with type 2 diabetes mellitus for more than 10 years on oral anti-diabetic drugs fulfilling our eligibility criteria were enrolled in our study. Blood samples for fasting blood sugar and fasting C-peptide level were taken before breakfast and uptake of anti-diabetic drugs. Correlation analysis was performed to evaluate the relationship between fasting C-peptide and glycaemic control with respect to glycated haemoglobin (HbA1c).

Results: Of the patients, 19.40% had insufficient beta cell reserve serum levels (C-peptide < 0.5 ng/ml), of which most of the patients (14/26 = 53.85%) had poor glycaemic control (HbA1c < 8.0%). Overall, there was a significant correlation between poor glycaemic control with respect to HbA1c and low serum C-peptide levels (p < 0.05). We found a significant association of beta cell dysfunction (low fasting C-peptide level) with the use of insulin secretagogue. The proportion of patients with C-peptide levels less than 0.5 ng/ml was lower in patients using sodium-glucose cotransporter-2 (SGLT-2) inhibitors as compared to insulin secretagogue.

Conclusion: SGLT-2 inhibitors should be preferred over other anti-diabetic drugs as an add-on to existing metformin therapy. Insulin requirement must be assessed in patients who have long-term type 2 diabetes mellitus.

## Introduction

Diabetes mellitus is a chronic metabolic and endocrine disorder characterized by anomalies in protein, lipid, and carbohydrate metabolism caused by deficits in insulin action or production. Persistent hyperglycaemia is another hallmark of the disease. It is a leading global cause of both morbidity and mortality. Globally, this illness is becoming more common [1].

The beta cells in the pancreatic islet synthesize proinsulin, which is a single polypeptide chain that combines human insulin and C-peptide. Proinsulin is cleaved by proteases to create equimolar amounts of mature insulin and C-peptide, which are then injected into the portal vein. The C-peptide, which has a molecular mass of 30200 g/mol, is a single peptide chain made up of 31 amino acids. Because it joins both the A and B chains of insulin in proinsulin, it is known as the C-peptide [2].

Obesity, pancreatic beta cell dysfunction, and insulin resistance are intricate pathophysiological pathways linked to the advancement of type 2 diabetes. Because both pathogenic states cause hyperglycaemia, the need for insulin rises. Elevated plasma glucose levels continue because there is inadequate glucose sensing to trigger insulin release, which leads to beta cell failure. As a result of this process, plasma glucose concentrations rise above the physiological level, causing insulin resistance to develop and hyperglycaemia to appear. Consequently, beta cells adapt to insulin resistance by secreting excessive amounts of insulin, which eventually results in the failure of β-cells [3]. In the development and progression of type 2 diabetes mellitus, β-cell dysfunction occurs after insulin resistance. Both pathogenic conditions interact with one another and probably exacerbate diabetes in a cumulative manner [3].

While insulin continues to be the mainstay of therapy in type 1 diabetes mellitus, its use in type 2 diabetes mellitus is limited to the presence of comorbidities such as chronic kidney disease, infection, and surgery [4]. Despite poor glycaemic control, there is a practice of increasing the dose of oral anti-diabetics or adding more drugs to the regimen due to the common perception that low endogenous insulin secretion is related to type 1 diabetes mellitus only and patient’s poor compliance with injectables [5].

As long-term insulin resistance could lead to beta cell dysfunction, there is a need to know the prevalence of low insulin production in patients with long-term diabetes mellitus [6]. C-peptide as a marker of insulin requirement is commonly measured in patients with type 1 diabetes mellitus, but low endogenous insulin production commonly remains unchecked in most patients with type 2 diabetes mellitus [7].

The American Association of Clinical Endocrinologists (AACE) clinical practice guidelines have suggested the addition of insulin therapy on entry glycated haemoglobin (HbA1c) level of 9.0% or failure to achieve glycaemic control after three months of therapy of triple oral anti-diabetic drug regimen [8]. However, the estimation of endogenous insulin production as an indication of starting insulin therapy in poor glycaemic control (HbA1c > 8.0%) is not mentioned in any clinical guidelines [9].

There is a scarcity of research that has correlated low endogenous insulin production measured by serum C-peptide level with poor glycaemic control with oral anti-diabetic drugs in patients with long-term type 2 diabetes mellitus in India (especially in Bihar). It is essential to check whether oral anti-diabetic drugs have any effect on beta cell function and how much low C-peptide level is related to glycaemic control. Keeping this perspective in mind, this study was conducted to assess the prevalence of beta cell dysfunction and low endogenous insulin release diagnosed by low serum C-peptide levels and its correlation with poor glycaemic control as per serum HbA1c, fasting, and post-prandial blood glucose level in patients with type 2 diabetes mellitus on oral anti-diabetic drugs.

## Materials and methods

This was a comparative observational and prospective study conducted in the outpatient department of endocrinology in a tertiary care centre in eastern India from January 2023 to June 2023. The study was started after the approval of the Institutional Ethics Committee (letter no: 86/IEC/IGIMS/2021) and written informed consent was obtained from the study participants before data collection, which was done under the principle of good clinical practice, taking care of the rights and confidentiality of study participants.

Eligibility criteria

Patients of either gender with an age between 18 and 60 years with type 2 diabetes mellitus and a duration greater than 10 years on the same oral anti-diabetic drugs for more than one year were included in our study. Patients with HbA1c greater than 9.0% or diagnosed with chronic kidney disease or active infection or with indications for any surgical procedures or any indication of the requirement of insulin therapy were excluded from our study.

Sampling method

Consecutive sampling was used, and each patient with long-term type 2 diabetes mellitus fulfilling our eligibility criteria during the study period was included in our study.

Methodology

Baseline demographic and clinical characteristics such as age, gender, duration of diabetes, comorbidities, and oral anti-diabetic drugs being taken were recorded in a proforma.

Blood pressure, waist circumference, height, and body weight were measured. Fasting blood sugar, HbA1c, fasting C-peptide, total cholesterol, triglyceride, low-density lipoprotein (LDL) cholesterol, and high-density lipoprotein (HDL) cholesterol concentrations were measured in the central hospital laboratory from venous blood samples.

In this study, fasting C-peptide concentrations of less than 0.5 ng/mL were considered insufficient, between 0.5 and 2 ng/mL as borderline, and more than 2 ng/mL as normal [10]. The groups were compared according to their demographic, comorbidity, and treatment characteristics, and anthropometric and biochemical data. Correlation analysis was performed to evaluate the relationship between fasting C-peptide and glycaemic control with respect to HbA1c.

Poor glycaemic control was defined as an HbA1c level greater than 8.0%, an HbA1c less than 7.5% was considered as good glycaemic control, and an HbA1c level between 7.5 and 8.0% was considered as fair glycaemic control [9]. Patients with HbA1c < 7.5% (good glycaemic control) acted as the control group.

Body weight, waist circumference, and height were measured by the same physician using standard measuring instruments. The World Health Organization defines waist circumference as the narrowest point on the waist with a small expiration halfway below the lowest rib and above the iliac crest. This measurement was taken while the patient was standing. The measurement of hip circumference was taken at the greater trochanter's largest circumference. Weight (in kg) divided by height (in m2) yields the body mass index (BMI). Blood pressure was obtained using an appropriate mercury sphygmomanometer (based on Korotkoff phase I and phase V sounds) on both arms with the patient in a comfortable sitting position after at least a 10-minute rest. The second measurement was performed on the arm with a higher value. Two measurements were taken at least three minutes apart and averaged to provide the values for systolic and diastolic pressure [11].

Fasting blood sugar levels were determined using glucose-oxidase peroxidase using Beckman Coulter 700AU (Brea, CA). A Tosoh HLC-723 G8 (Tosoh G8, variant-mode) ion-exchange high-performance liquid chromatography (HPLC) system (Tosoh, Tokyo, Japan) was used for HbA1c measurements. The C-peptide was measured using a chemiluminescence microparticle immunoassay in the Abbott Architect I2000 autoanalyzer (Abbott Laboratories, Abbott Park, IL). Fasting plasma, total cholesterol, HDL and LDL cholesterol, and triglyceride concentrations were determined using spectrophotometry based on Beer-Lambert law by the Beckman Coulter 5800AU machine (Brea, CA). Blood samples for fasting blood sugar and fasting C-peptide level were taken before breakfast and uptake of anti-diabetic drugs.

Statistical analysis

Data collected were presented in tabular form using Microsoft Excel 365 (Microsoft Corporation, Redmond, WA) and then transferred to SPSS version 24 (IBM Corp., Armonk, NY) for further statistical analysis. Categorical data such as the number and percentage of patients with respect to C-peptide level, HbA1c level, and type of anti-diabetic regimens were evaluated using the chi-square test for determining the statistical significance of the difference between groups. An unpaired t-test was used to compare continuous data such as age, BMI, waist circumference, blood pressure, and fasting or post-prandial blood glucose level with a p-value of less than 0.05 as a measure of statistical significance.

## Results

A total of 134 patients with type 2 diabetes mellitus for more than 10 years on oral anti-diabetic drugs fulfilling our eligibility criteria were enrolled in our study. They were grouped according to C-peptide and HbA1c levels, as shown in Table 1.

**Table 1:** Distribution of patients with respect to serum C-peptide and HbA1c levels HbA1c: glycated haemoglobin.

Fasting serum C-peptide level	Total (n, %)	HbA1c level			P-value (chi-square)
		<7.5% (n, %)	7.5-8.0% (n, %)	>8.0% (n, %)	
<0.5 ng/ml	26 (19.40)	2 (6.90)	10 (16.13)	14 (32.56)	0.0074 (chi-square value = 13.96)
0.5-2.0 ng/ml	49 (36.57)	8 (27.59)	23 (37.10)	18 (41.86)	
>2.0 ng/ml	59 (44.03)	19 (65.52)	29 (46.77)	11 (25.58)	
Total	134	29	62	43	

Patients with HbA1c < 7.5% (good glycaemic control) acted as the control group. There was no statistically significant difference in common confounding factors such as age, gender, diet, duration of diabetes, obesity, compliance to anti-diabetic therapy, and concomitant medications between different categories of patients (as per C-peptide level or HbA1c level or anti-diabetic regimen).

**Figure 1:**
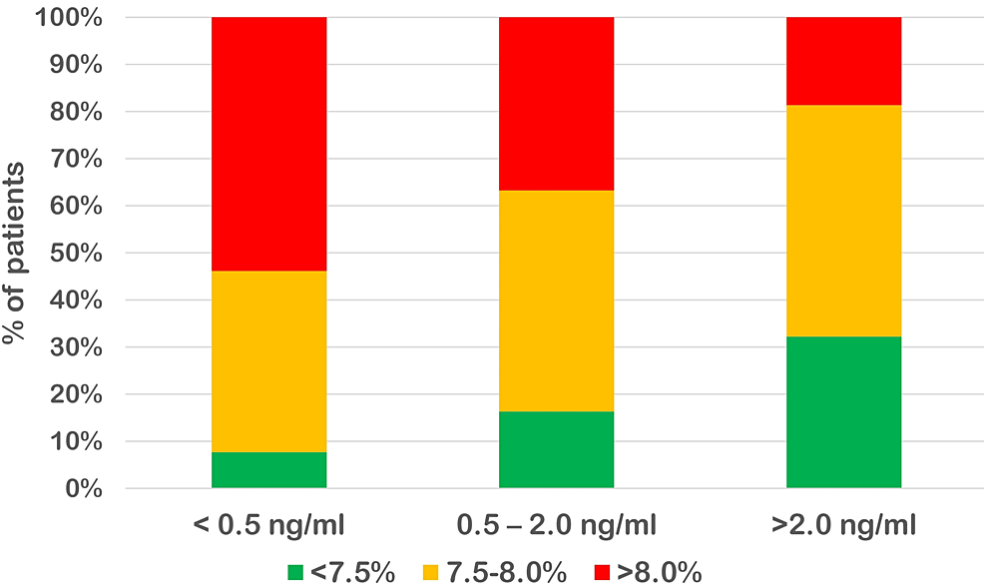
Correlation of HbA1c level with respect to C-peptide level HbA1c: glycated haemoglobin.

Of the patients, 26 (19.40%) had serum levels of C-peptide < 0.5 ng/ml, indicating insufficient beta cell reserve. Of these, 14 (53.85%) patients had poor glycaemic control (HbA1c > 8.0%). Only 11 (25.58%) patients with HbA1c > 8.0% had sufficient beta cell reserves (C-peptide > 2.0 ng/ml) compared to 19 (65.52%) patients with good glycaemic control (HbA1c < 7.5). Overall, there was a significant correlation between poor glycaemic control with respect to HbA1c and low serum C-peptide levels (p < 0.05) (Table 1). The difference in the three groups with respect to HbA1c can be clearly appreciated in Figure 1.

We found no correlation of poor glycaemic control C-peptide level with age, sex, blood pressure, waist, and hip circumference (p > 0.05). There was slight female preponderance in patients with sufficient, insufficient, or borderline beta cell reserves. Patients with insufficient beta cell reserves had a significantly higher duration of diabetes as compared to patients with sufficient or borderline beta cell reserves (p < 0.001) (Table 2).

**Table 2:** Comparison of demographic and clinical characteristics among patients with different C-peptide levels SD: standard deviation; BMI: body mass index; SBP: systolic blood pressure; DBP: diastolic blood pressure.

Variables	C-peptide level			P-value
	<0.5 ng/ml (n =26)	0.5-2.0 ng/ml (n = 49)	>2.0 ng/ml (n = 59)	
Age in years (mean ± SD)	63.88 ± 8.67	61.40 ± 9.18	62.09 ± 8.91	0.52 (F = 0.66)
Female gender, n (%)	14 (53.85)	26 (53.06)	31 (52.54)	0.99 (chi-square value = 0.01)
Duration of diabetes in years (mean ± SD)	14.76 ± 1.73	14.27 ± 2.01	12.31 ± 1.12	<0.0001 (F = 29.51)
BMI in kg/m^2^ (mean ± SD)	25.49 ± 3.03	26.44 ± 2.29	24.57 ± 2.33	0.0007 (F = 7.71)
Waist circumference (mean ± SD)	94.78 ± 9.16	96.67 ± 9.88	95.51 ± 10.43	0.71 (F = 0.34)
Hip circumference (mean ± SD)	102.92 ± 11.39	105.28 ± 10.98	103.79 ± 12.04	0.66 (F = 0.41)
SBP in mmHg (mean ± SD)	143.53 ± 16.77	142.56 ± 19.64	143.25 ± 20.27	0.97 (F = 0.03)
DBP in mmHg (mean ± SD)	81.58 ± 8.31	80.13 ± 8.46	82.65 ± 7.73	0.28 (F = 1.29)

**Figure 2:**
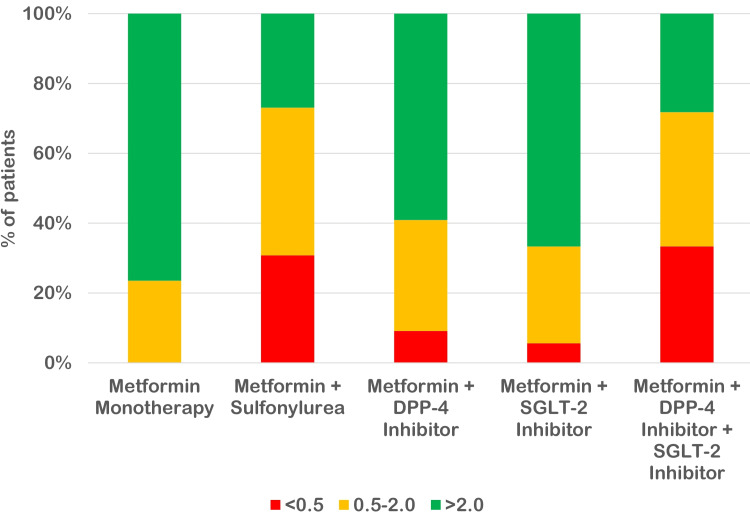
Association of C-peptide level with anti-diabetic drug regimens DPP-4: dipeptidyl peptidase-4; SGLT-2: sodium-glucose cotransporter-2.

The use of metformin + sulfonylurea was associated with a low C-peptide level. Next to sulfonylurea, the use of dipeptidyl peptidase-4 (DPP-4) inhibitor was more frequently found in patients with low C-peptide levels as compared to metformin + sodium-glucose cotransporter-2 (SGLT-2) inhibitor therapy. However, utilization of the metformin + SGLT-2 inhibitor regimen was lower as compared to other regimens. Overall, we found a significant association of beta cell dysfunction (low fasting C-peptide level) with the use of insulin secretagogue (p < 0.05) (Table 3). This association can be clearly anticipated in Figure 2.

**Table 3:** Association of C-peptide level with anti-diabetic drug regimens DPP-4: dipeptidyl peptidase-4; SGLT-2: sodium-glucose cotransporter-2.

Variables	C-peptide level			P-value
	<0.5 ng/ml (%, n =26)	0.5-2.0 ng/ml (%, n = 49)	>2.0 ng/ml (%, n = 59)	
Metformin monotherapy	0 (0.00)	4 (8.16)	13 (18.84)	0.0009 (chi-square value = 26.44)
Metformin + sulfonylurea	8 (30.77)	11 (22.45)	7 (10.15)	
Metformin + DPP-4 inhibitor	4 (15.38)	14 (28.57)	26 (37.68)	
Metformin + SGLT-2 inhibitor	1 (3.85)	5 (10.20)	12 (17.39)	
Metformin + DPP-4 inhibitor + SGLT-2 inhibitor	13 (50.00)	15 (30.61)	11 (15.94)	

We did not find any significant correlation between C-peptide level or glycaemic control with fasting plasma, total cholesterol, HDL and LDL cholesterol, and triglyceride concentrations (p > 0.05).

Low serum C-peptide levels had a significant correlation with poor glycaemic control with respect to fasting and post-prandial blood glucose levels (Table 4).

**Table 4:** Association of C-peptide level with fasting and post-prandial blood glucose level SD: standard deviation.

Fasting serum C-peptide level	Fasting blood sugar (mean ± SD)	Post-prandial blood sugar (mean ± SD)
<0.5 ng/ml	154.41 ± 13.75	236.73 ± 16.92
0.5-2.0 ng/ml	149.96 ± 12.53	211.32 ± 16.38
>2.0 ng/ml	141.39 ± 7.24	197.18 ± 10.07
P-value (one-way ANOVA)	<0.0001 (F = 15.89)	<0.0001 (F = 71.67)

## Discussion

In this observational study, there was a significant correlation between low serum C-peptide levels and poor glycaemic control with respect to HbA1c and fasting blood sugar levels. There was a slight female preponderance in patients with sufficient, insufficient, or borderline beta cell reserves. Patients with insufficient beta cell reserves had a significantly longer duration of diabetes as compared to patients with sufficient or borderline beta cell reserves.

A varied combination of hepatocellular and peripheral insulin resistance, as well as pancreatic beta cell failure, is the cause of hyperglycaemia in type 2 diabetes. Though at varying rates of advancement, the primary cause of the progression of hyperglycaemia is the ongoing decrease of beta cell activity [12].

The presence of a malfunctioning beta cell predates the onset of hyperglycaemia, as evidenced by elevated levels of proinsulin in isolation as well as proinsulin/C-peptide or proinsulin/insulin ratios in prediabetic individuals [13-15].

Interestingly, when C-peptide is employed as the denominator for proinsulin ratio computations, it may be a stronger indicator of unstable beta cells and a better predictor of the development of type 2 diabetes than insulin because it is unaffected by variability in hepatic clearance of insulin [16,17]. A progressive decrease in plasma C-peptide is observed following the development of type 2 diabetes. Despite this, whether a person is taking a basal-bolus regimen or basal insulin alone, it could remain detectable for over two decades, even in those who have been switched to insulin therapy [18].

According to a few studies, C-peptide may also be helpful in understanding the variability of type 2 diabetes and may offer helpful recommendations for the safe beginning and adjustment of basal insulin. C-peptide has been substituted for insulin in the homeostasis model assessment (HOMA2) B calculation to identify individuals with severe insulin-deficient type 2 diabetes (SIDD) [19]. Compared to previous groups of adult-onset diabetes, this group of individuals had the greatest familial risk score for type 2 diabetes, the lowest level of metabolic control throughout time, a faster development towards continuous insulin use, and a greater likelihood of diabetic retinopathy [19,20].

We discovered a significant association between the use of insulin secretagogue medications, such as DPP-4 inhibitors and sulfonylureas, and beta cell dysfunction, which is characterized by a low fasting C-peptide level.

The decrease of beta cell mass in patients with type 2 diabetes mellitus has been compellingly demonstrated by recent investigations [21,22]. Therefore, medications that accelerate beta cell death could have negative long-term effects and increase the need for insulin. It was demonstrated in Maedler et al.'s study that glibenclamide, a sulfonylurea, caused beta cell death in human islets. Although this effect was only seen in vitro, it might still have therapeutic significance because the plasma concentrations that had significant results in their studies fell within the range that is generally found in patients receiving regular medication [23].

Measurements of C-peptide may also be useful in predicting how insulin-treated patients will respond to glucagon-like peptide-1 receptor agonist (GLP-1 RA) therapy, according to a large observational and prospective study. Patients demonstrated a 0.4% reduction in HbA1c following six months of GLP-1 RA therapy for every 1 nmol/L decrease in fasting C-peptide. However, in patients who were not receiving insulin, C-peptide levels were not linked to therapeutic response in the same study [24].

C-peptide testing might play a part in helping patients choose their insulin dosage in the future. When type 2 diabetes is treated with insulin, many of these people are able to maintain good glycaemic control with just intermediate or long-acting insulin. However, if the disease worsens, fast-acting insulin during meals may become necessary. In both type 1 and type 2 diabetes, C-peptide is negatively correlated with glycaemic variation and rise in plasma glucose after meal. In an experimental setting, it is also inversely correlated with the response to prandial insulin in a group of individuals with diabetes [25-27]. Absolute insulin deficit and need are confirmed by stimulated blood C-peptide less than 0.2 nmol/l [28].

A small sample size could be a limitation of this study. However, the results strongly highlight lesser prescription of insulin therapy to patients with uncontrolled type 2 diabetes and with limited endogenous production of insulin in eastern India.

## Conclusions

The study shows a significant prevalence of beta cell dysfunction in patients with type 2 diabetes in eastern India. The low beta cell function marked by low C-peptide levels was associated with a poor response to oral anti-diabetic drugs such as sulfonylureas. A cheap, accessible test that can help with diabetes clinical care is C-peptide measurement, especially for individuals who are not on insulin and whose response to oral anti-diabetic drugs is unsatisfactory. SGLT-2 inhibitors should be preferred over other anti-diabetic drugs as an add-on to existing metformin therapy. Insulin requirements must be assessed in patients with long-term type 2 diabetes mellitus.
